# Complete genome sequence, phenotypic correlation and pangenome analysis of uropathogenic *Klebsiella* spp

**DOI:** 10.1186/s13568-024-01737-w

**Published:** 2024-07-04

**Authors:** Abhirami Krishnamoorthy Sundaresan, Jaya Gangwar, Aravind Murugavel, Ganesh Babu Malli Mohan, Jayapradha Ramakrishnan

**Affiliations:** 1 Actinomycetes Bioprospecting Lab, Centre for Research in Infectious Diseases (CRID), School of Chemical and Biotechnology (SCBT), SASTRA Deemed University, Tirumalaisamudram, Thanjavur, 613401 Tamil Nadu India; 2 Microbial Omics Lab, Centre for Research in Infectious Diseases (CRID), School of Chemical and Biotechnology (SCBT), SASTRA Deemed University, Tirumalaisamudram, Thanjavur, 613401 Tamil Nadu India; 3 Center for Tropical and Emerging Global Diseases (CTEGD), University of Georgia, Georgia Athens, United States of America

**Keywords:** Pangenome, Genotype-Phenotype association, *Klebsiella spp*.,, Uropathogen, Hypervirulence

## Abstract

Urinary tract infections (UTI) by antibiotic resistant and virulent *K. pneumoniae* are a growing concern. Understanding the genome and validating the genomic profile along with pangenome analysis will facilitate surveillance of high-risk clones of *K. pneumoniae* to underpin management strategies toward early detection. The present study aims to correlate resistome with phenotypic antimicrobial resistance and virulome with pathogenicity in *Klebsiella* spp. The present study aimed to perform complete genome sequences of *Klebsiella* spp. and to analyse the correlation of resistome with phenotypic antimicrobial resistance and virulome with pathogenicity. To understand the resistome, pangenome and virulome in the *Klebsiella* spp, the ResFinder, CARD, IS Finder, PlasmidFinder, PHASTER, Roary, VFDB were used. The phenotypic susceptibility profiling identified the uropathogenic kp3 to exhibit multi drug resistance. The resistome and in vitro antimicrobial profiling showed concordance with all the tested antibiotics against the study strains. Hypermucoviscosity was not observed for any of the test isolates; this phenotypic character matches perfectly with the absence of *rmpA* and *magA* genes. To the best of our knowledge, this is the first report on the presence of *ste, stf, stc* and *sti* major fimbrial operons of *Salmonella enterica* serotype Typhimurium in *K. pneumoniae* genome. The study identifies the discordance of virulome and virulence in *Klebsiella* spp. The complete genome analysis and phenotypic correlation identify uropathogenic *K. pneumoniae* kp3 as a carbapenem-resistant and virulent pathogen. The Pangenome of *K. pneumoniae* was open suggesting high genetic diversity. Diverse K serotypes were observed. Sequence typing reveals the prevalence of *K. pneumoniae* high-risk clones in UTI catheterised patients. The study also highlights the concordance of resistome and in vitro susceptibility tests. Importantly, the study identifies the necessity of virulome and phenotypic virulence markers for timely diagnosis and immediate treatment for the management of high-risk *K. pneumoniae* clones.

**Supplementary Information:**

The online version contains supplementary material available at 10.1186/s13568-024-01737-w.

## Introduction

Urinary Tract Infections (UTIs) are commonly treatable, however complicated UTIs associated with indwelling devices, immunosuppression and urinary tract abnormalities are highly challenging to treat (Cristea et al. 2017; Liu et al. 2020). Worldwide 20–25% of complicated UTIs are due to indwelling catheters during hospitalization (Kranz et al. 2020). The microbiology of acute and chronic catheterization is varied. For instance, *E.coli*, *Enterococcus* spp., coagulase-negative *Staphylococcus* spp., *Pseudomonas aeruginosa* and *Klebsiella pneumoniae* are frequently isolated from acute infections and *Proteus mirabilis* is uniquely associated with chronic indwelling catheters (Niveditha et al. 2012). Multidrug resistance has been increased all over the world which is considered a public health threat. Several recent investigations reported the emergence of multidrug-resistant bacterial pathogens from different origins that increase the necessity for the proper use of antibiotics. Besides, the routine application of antimicrobial susceptibility testing to detect the antibiotic of choice as well as the screening of the emerging MDR strains (Algammal et al. 2019, 2022a, 2022b, 2024; Kareem et al. 2021; Elbehiry et al. 2022; Shafiq et al. 2022). *K. pneumoniae* is gaining prominent attention in clinical settings as it has a large accessory genome that determines the pathotypes, hypervirulent and classical (Holt et al. 2015; Thomas and Russo 2019). They frequently cause life threatening nosocomial infections and invasive infections like endophthalmitis, meningitis and liver abscesses. When the infection is established by *Klebsiella* spp. polymorphonuclear granulocytes and serum complement proteins are employed at the infection site as a first line of defense mechanism. *Klebsiella* spp. capsule is made up of complex acidic polysaccharides which enables the species to escape from the host defense mechanism. Other than capsules, they have many fimbrial and non-fimbrial adhesions which allow the pathogen to adhere to the host cells establishing its colonisation in the host. The virulence factors of *K. pneumoniae* are harboured in both the core and accessory genomes (Martin and Bachman 2018). This helps to identify the pathotype of *K. pneumoniae* from two closely related species, *K. variicola* and *K. quasipneumoniae*. *K. variicola* is another emerging pathogen that colonises humans (Rodríguez-Medina et al. 2019). Multidrug resistance and ESBL producing *K. variicola* isolates were found to cause UTIs and Bloodstream Infection (BSI) leading to increased mortality (Maatallah et al. 2014). *K. variicola* is also associated with neonatal sepsis and has similar virulence factors harboured by *K. pneumoniae*. The misidentification of *K. variicola* as *K. pneumoniae* was reported frequently (Humberto Barrios-Camacho, Alejandro Aguilar-Vera et al. 2019; Rodríguez-Medina et al. 2019). The routine traditional culture techniques fail to discriminate the major species within the complex.

The virulence factors associated with *K. pneumoniae* are Capsular Polysaccharide (CPS), lipopolysaccharides, type I and III fimbriae, type IV pili, siderophores, type IV secretion system and allantoin utilization (Zhu et al. 2021). These virulence factors work together in promoting the biofilm forming ability of *Klebsiella* spp. Type I and III fimbriae interact with the host by adhering to the cells or the surface of the indwelling devices. Type IV pili are involved in host pathogen adherence. Siderophores play an important role in augmenting virulence by promoting growth and biofilm formation. CPS is the major virulence factor that helps to protect the bacteria and in the formation of biofilm mainly on the indwelling device causing severe infections (Schembri et al. 2005; Paczosa and Mecsas 2016; Guerra et al. 2022).

Pandrug resistant *K. pneumoniae* is frequently been reported in recent years and is associated with BSI. They are the leading cause of secondary infections in Intensive care unit (ICU) patients. Treatment of pandrug resistant *K. pneumoniae* is difficult, and combination therapy is used in such patients (Coskun and Atici 2020; Sundaresan et al. 2022). The identification of drug-resistant and virulent pathogens is crucial for rapid diagnosis and treatment. Multiplex polymerase chain reaction techniques can identify the presence of particular Antimicrobial Resistance genes (ARGs) and virulence genes, but they cannot detect resistance owing to mutational evolution (Anjum et al. 2017).

Next-generation sequencing and analysis could potentially replace existing microbiological procedures by providing species identification, tracking of Antimicrobial Resistance (AMR) patterns, epidemic tracking to physicians, scientists, and public health specialists (Petti 2007; Lefterova et al. 2015; Punina et al. 2015). Our earlier study using publicly accessible genomes of 153 *K. pneumoniae* Indian isolates, the majority of the isolates source were from BSI, offered unique genetic insights for identifying strains that require rapid treatment and prevention of dissemination in clinical settings (Sundaresan et al. 2022).

This work aims to monitor the concordance of genotypic-phenotypic AMR along with the virulence profiling of *K. pneumoniae*. The study also focuses on genetic diversity, mechanisms of resistance, and virulence in uropathogenic *K. pneumoniae*. As *K. variicola* is frequently misdiagnosed as *K. pneumoniae* in clinical settings, the present study used both *K. pneumoniae* and *K. variicola*. The genomes were sequenced using Illumina and Nanopore followed by phenotypic antibiotic susceptibility tests. Furthermore, in vitro virulence characterisation using string test, biofilm formation and validation of virulence in Zebrafish in vivo model were performed. Such a thorough strategy will provide insights into the management techniques that will underpin accurate early diagnosis and clinical attention.

## Materials and methods

### Strains

The *K. pneumoniae* (*n* = 3) strains were obtained from Dr. D. Y. Patil Medical College, Pune, India sourced from the urine of the UTI infected patients. The presumptive identification of the isolates was performed using HiCrome UTI agar medium (Cheepurupalli et al. 2017). The environmental strain *K. variicola* MTCC 4030 was purchased from the Microbial Type Culture Collection, India.

### Antibiotics and other reagents

UTI agar medium, Crystal violet, antibiotics (Ampicillin, Oxacillin, Methicillin, Cefazolin, Cefadroxil, Cefuroxime, Cefepime, Cefpodoxime, Ceftazidime, Ertapenem, Imipenem, Ciprofloxacin, Erythromycin, Gentamycin, Chloramphenicol, Colistin) and biochemical reagents were purchased from Himedia, India.

### Identification using biochemical test

For the identification of the isolates, biochemical tests such as indole production, urease test, MR-VP test and lactose fermentation were performed (Podschun et al. 1998; Podschun et al. 2001).

### DNA quality assessment

The concentration and purity of genomic DNA were quantified using the Nanodrop Spectrophotometer. The integrity of the DNA was observed by agarose gel electrophoresis. DNA concentration was assessed with Qubit dsDNA HS assay kit. The strain purity of the samples was checked by 16 S rRNA gene sequencing. PCR amplification was performed with 30–50 ng of the genomic DNA as a template and using 16 S rDNA primers (27forward and 1492Reverse) and Takara ExTaq in a 25 µl reaction mix. 1.5 kb PCR product was generated, purified and used for Sanger sequencing.

16 S rDNA Forward: AGAGTTTGATCCTGGCTCAG − 20 mer.

16 S rDNA Reverse: TACGGCTACCTTGTTACGACTT − 22 mer.

The methods of Illumina and Nanopore library preparation and sequencing are provided in Supplementary Method S1.

### Genome assembly and annotation

The raw data obtained from the paired-end sequencing were quality checked with the FastQC tool (version 0.11.9) and *de novo* hybrid Illumina-Nanopore assemblies were generated using unicycler in Galaxy and PATRIC (The PathoSystems Resource Integration Center) (Wick et al. 2017). The annotation was performed using Prokka and RAST (Rapid annotation using subsystem technology).

### Bioinformatics analysis

The resistome, replicon types and virulome were identified using a BLAST-based approach. The Single Nucleotide Polymorphism (SNP) based evolutionary relationship of *K. pneumoniae* and *K. variicola* with other *Klebsiella* species were predicted using CSI Phylogeny with a bootstrap value of 1000 and analysed using Figtree (Kaas et al. 2014). A comprehensive AMR analysis by CARD (Comprehensive Antibiotic Resistance Database) (McArthur et al. 2013) and ResFinder was performed with a 90% threshold for % ID. Gene sequence with 100% identity is confirmed for its presence in the genome (Feng et al. 2021). For plasmid replicons, PlasmidFinder (Carattoli et al. 2014), K and O loci by Kaptive database (Wyres et al. 2020), IS (Insertional Sequence) elements by IS finder (Siguier et al. 2006) were used. For the identification of prophages within each genome PHASTER and Virus-Host DB were used (Marques et al. 2021); Bleriot et al. 2020). For virulome analysis, the CDSs were identified using VFanalyser available in VFDB (Virulence Factor Database) (Kwon et al. 2016). Multilocus Sequence Typing (MLST) data for the 4 genomes were collected from https://pubmlst.org (Jolley et al. 2018). The seven housekeeping genes *gapA, infB, mdh, pgi, phoE, rpoB* and *tonB* of *Klebsiella* spp. were used to construct the phylogenetic tree based on MLST. The comprehensive results of resistome, virulome and episome were consolidated using the interactive Tree of Life (iTOL) (Letunic and Bork 2016).

## Pangenome

Pangenome analysis was performed using Roary with a gff annotation file produced by Prokka genome annotation (Page et al. 2015). The data was analysed and viewed using Phandango and R version 4.1.0 (Hadfield et al. 2018). Reference strain was selected based on the ST of the study strains.

### AMR phenotypic characterization

Disk diffusion and MIC for various classes of antibiotics were performed according to Clinical and Laboratory Standards Institute guidelines which include β-lactams (Ampicillin, Oxacillin, Methicillin, Cefazolin, Cefadroxil, Cefuroxime, Cefepime, Cefpodoxime, Ceftazidime, Ertapenem, Imipenem), aminoglycosides (Gentamycin), fluoroquinolones (Ciprofloxacin), macrolide (Erythromycin), chloramphenicol and polymyxin (Colistin) (Cheepurupalli et al. 2017). MIC was performed for all the antibiotics with concentrations from 0.5 to 8 µg/mL and inoculated with 0.5 McFarland of bacterial suspension. Further, the plates were incubated at 37^0^ C for 24 h and the OD values were measured at 600 nm. Similarly, for the disk diffusion method to the Muller Hinton agar plates, the antibiotic disks were placed after swabbing the plate with the same concentration of bacterial suspension. After the incubation period, the Zone of sensitivity were measured.

### Multiple antibiotic resistance (MAR) indexing

The high-risk isolates can be determined by using MAR indexing. Thus, the MAR index was determined according to Krumperman (1983). The MAR index was performed for a single isolate and also aggregate MAR index was calculated. For a single isolate MAR index is defined as a/b, where ‘a’ denotes the number of antibiotics to which the isolate was resistant and ‘b’ denotes the total number of antibiotics exposed to the isolate. To determine the aggregate MAR index, a/(b x c) is applied. Here, ‘a’ denotes the aggregate antibiotic resistance score of all isolates, ‘b’ denotes the number of antibiotics and ‘c’ denotes the total number of isolates (Titilawo et al. 2015).

### In vitro **and **in vivo **virulence correlation study**

#### Hypervirulence determination using string test

To check the hypermucoviscosity phenotype with the absence of the *rmpA* and *magA* gene, string test was performed. UTI agar, Luria Bertani and blood agar medium were used and observed for 3 days. A colony was stretched using an inoculation loop to observe a string of > 5 mm in length, which is defined as a positive string test (Shon et al. 2013).

#### Hypermucoviscosity determination using sedimentation method

Hypermucoviscosity was assessed using the sedimentation method. The overnight culture was pelleted at 10,000 RPM for 10 min. The pellet was resuspended in 1 ml PBS to an OD_600_ = 1.0. Samples were subjected to low-speed centrifugation at 1000 x *g* for 5 min. The cells remaining in the supernatant were quantified at OD_600_ (Mikei et al. 2021).

#### In vitro **biofilm formation and correlation with genome**

To correlate the predicted gene with proficiency of in vitro biofilm formation, a 96-well plate was used to form biofilm using 0.1% glucose as medium. The biofilm was estimated using Crystal Violet (CV) assay (Lalitha et al. 2017). The detailed procedure is provided in Supplementary Method 1.

#### Virulence validation in zebrafish

To validate the virulence/pathogenicity of kp1, kp2, kp3 and kp4, Zebrafish was used as an animal model. Zebrafish in length 4 to 5 cm and weighing 300 mg were procured from a local aquarium. To use animals for experiments, proper national and/or institutional guidelines (Animal biosafety level 2) were followed. The medium-sized Zebrafish in each group (*n* = 7) were infected intramuscularly with 10 µl of culture (10^12^ CFU/ml). The control group received 10 µl of PBS. The fish were monitored for 5 days for pathological changes such as superficial infection at the injection site, feed intake, mobility and survival. The dissected muscle tissue of the infected fish was used to estimate the pathogen load. For histopathology, the cut sections of the 5 mM specimen were stained with hematoxylin and eosin (Cheepurupalli et al. 2017).

#### Statistical analysis

The Kaplein Meier survival curve and the parametric test - One way ANOVA were plotted using GraphPad Prism (version 5.01).

## Results

Complete genome sequencing of *Klebsiella* spp. and its phenotypic correlation were performed and analysed to anticipate the concordance between genotype and phenotype. Pangenome analysis, AMR prediction, detection of IS elements, phages and serotypes have shed light on understanding the genome of *Klebsiella* spp.

### Phenotypic characteristics of the recovered isolates

The phenotypic methods showed that the four isolates were found to be Gram-negative, rod shaped and nonmotile. They are mucoid and can form biofilm. The isolates were indole negative, Methyl red negative and Voges-Proskauer positive. They are found to be lactose fermenters when cultured on Mackonkey agar and were able to hydrolyse urea.

### Complete genome analysis

In the present study, genomic analysis was performed for the uropathogenic *K. pneumoniae* (*n* = 3; kp1, kp2, kp3) and *K. variicola*, from the seed of wild rice (*n* = 1; kp4) [Supplementary Table S1 (Sheet 1)]. The Illumina sequencing of kp1, kp2, kp3 and kp4 generated 2,805,168, 2,570,036, 2,275,354 and 2,498,564 raw reads. The FastQC results showed good quality scores of raw Illumina reads. The nanopore sequencing generated 35,782, 52,004, 92,057 and 65,364 reads [Supplementary Table S1 (Sheet 2)]. The genome sequences of the isolates were deposited under BioProject accession number PRJNA650119.

The Complete Genome Sequencing (CGS) of kp1, kp2, kp3 (*K. pneumoniae*) and kp4 (*K. variicola*) comprised 5.1 Mbp, 6.1 Mbp, 5.6 Mbp and 5.8 Mbp respectively. The PATRIC server annotated hybrid genome of kp1, kp2, kp3 and kp4 comprised of protein-coding genes (5286, 6653, 5690, 5840), tRNA genes (88, 83, 89, 88) and rRNA genes (24, 22, 25, 25) respectively. The hybrid assembly showed a smaller number of contigs when compared with Illumina. The comparison of genome features of Illumina, Nanopore and hybrid genome sequences were performed using PATRIC [Supplementary Table S1 (Sheet 3)]. For further bioinformatic analysis, hybrid genome was used.

Rapid Annotations using the Subsystems Technology server (RAST) provided genome analysis of the set of proteins that together implement a specific biological process or structural complex (subsystem) (Supplementary Fig. S1). An average of 2,200 genes in each genome was classified as metabolism, protein processing, stress response, defence, virulence, membrane transport, cellular processes, regulation and cell signalling. The metabolic genes are predominant in all 4 complete genomes. According to the RAST server number of virulence genes was high (*n* = 69) in the kp2 genome.

### **Sequence typing reveals *K. pneumoniae *high-risk clones in UTI catheterised patients**

The Sequence Type (ST) of the 3 clinical isolates are kp1-ST200, kp2-ST45 and kp3-ST147. The ST of kp4 was not known. ST45 and ST147 were reported as high-risk hypervirulent clones associated with neonatal infections and nosocomial transmission carrying carbapenemases mainly *bla*_KPC−2_, *bla*_KPC−3_, *bla*_OXA−48_ and *bla*_NDM−1_ (Sands et al. 2021; Cienfuegos-gallet et al. 2022). ST147 was strongly associated with multidrug resistance (*bla*_NDM_, *bla*_KPC_, *bla*_OXA−48_, *bla*_OXA−181_, *bla*_VIMS_) and colonization in the host (Gondal et al. 2020; Peirano and Liang Chen, Barry N. Kreiswirth 2020). ST200 belongs to colistin resistance strains (Singh et al. 2017). Noteworthy, in the present study ST200 (kp1) and ST45 (kp2) strains were harboured with Class A SHV genes alone and colistin resistance was not detected. ST147 is majorly identified as a high-risk hypervirulent clone, found endemic in India, Italy, Greece and some North African countries (Huynh et al. 2020). ST147 (kp3) was found to be harboured with Class A, B and D enzymes with a high number of virulence genes. Based on STs, kp3 belongs to high-risk clones with broader AMR and virulence.

**Fig. 1:**
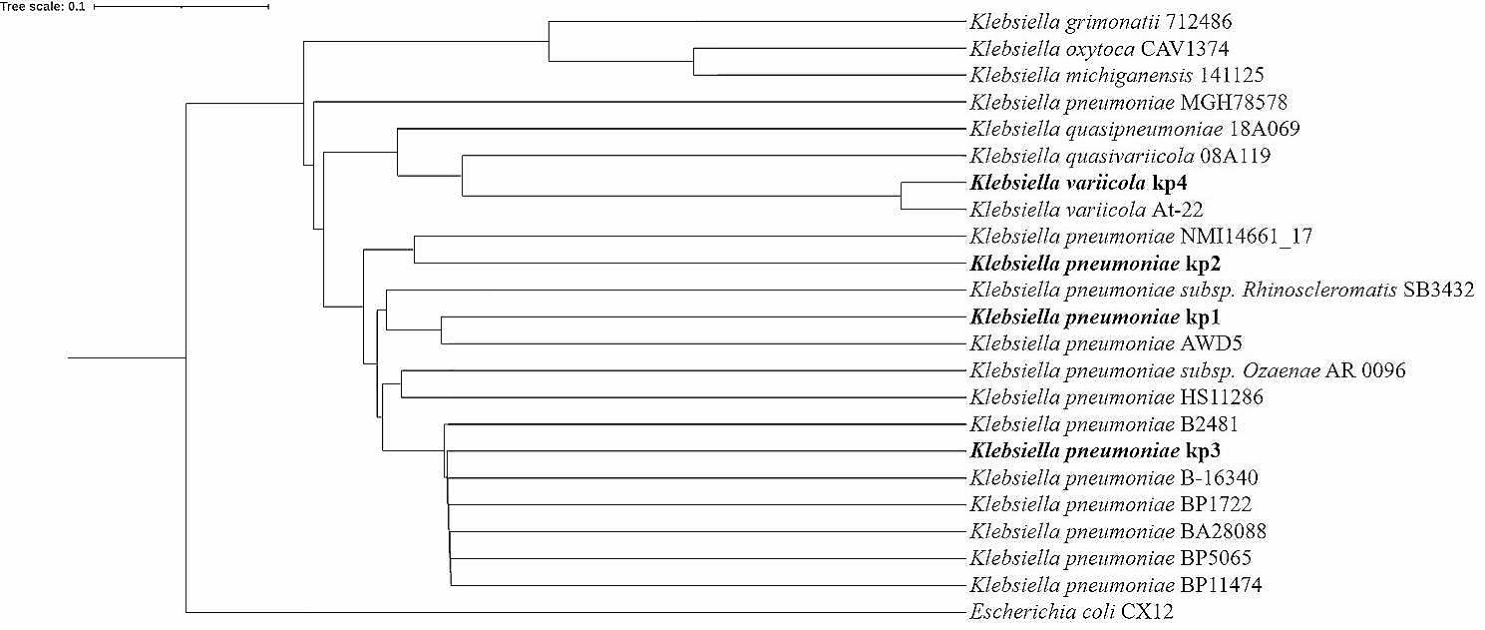
Evolutionary descent of study strains: SNP based phylogenetic tree, showing the relationship between the study strains and other *Klebsiella* spp

For phylogenetic relations, the genomes of the isolates were screened with a broader context including related *Klebsiella* spp. The tree was constructed based on SNP and mapped with *E. coli, K. oxytoca, K. michiganensis, K. grimontii, K. variicola, K. quasivariicola, K. quasipneumoniae, K. ozaenae, K. rhinoscleromatis*. The SNP tree considers 100% of the chromosomal genome. The environmental reference strain *K. pneumoniae* genome (AWD5) and clinical *K. pneumoniae* with ST200, ST45 and ST147 sourced from PATRIC were used to analyse its relationship with study genomes. The midpoint was divided into 2 major clades. Clade 1 represents kp4 closely related to *K. variicola.* Other clinical strains namely kp1, kp2 and kp3 along with its reference strains were grouped in the same clade. *E. coli* is out grouped in clade 2. The SNP analysis confirmed that the kp1, kp2 and kp3 genomes belong to *K. pneumoniae* and the kp4 genome belongs to *K. variicola* (Fig. 1). To study the evolutionary descent of the study strains among the publicly available *K. pneumoniae* genome, SNP based phylogenetic tree was constructed (Supplementary Fig. S2).

### **Diverse K serotypes found in*Klebsiella*spp.**

Out of the 4 isolates analysed, 3 strains were identified with novel gene clusters of K serotypes. The isolates have less coverage identity with the existing serotype and thus the isolates could be putatively assigned to novel serotypes, suggesting the high diversity of capsular polysaccharides in *Klebsiella* spp. The isolates kp1, kp2, kp3 and kp4 have high sequence homology with KL58 (99%), KL7 (65%), KL107 (55%) and KL50 (62%) respectively. Except for the kp1 isolate, all others were identified to have poor match confidence with the available sequences in the database. In the case of O serotype, the strains kp1, kp3 and kp4 strains were found to have sequence homology (100%) with O3b, O3/O3a and O3a serotypes respectively. The serotype analysis concludes the diversity of K serotypes in *Klebsiella* spp.

### **Pangenome identifies high genetic diversity in*Klebsiella*spp.**

**Fig. 2:**
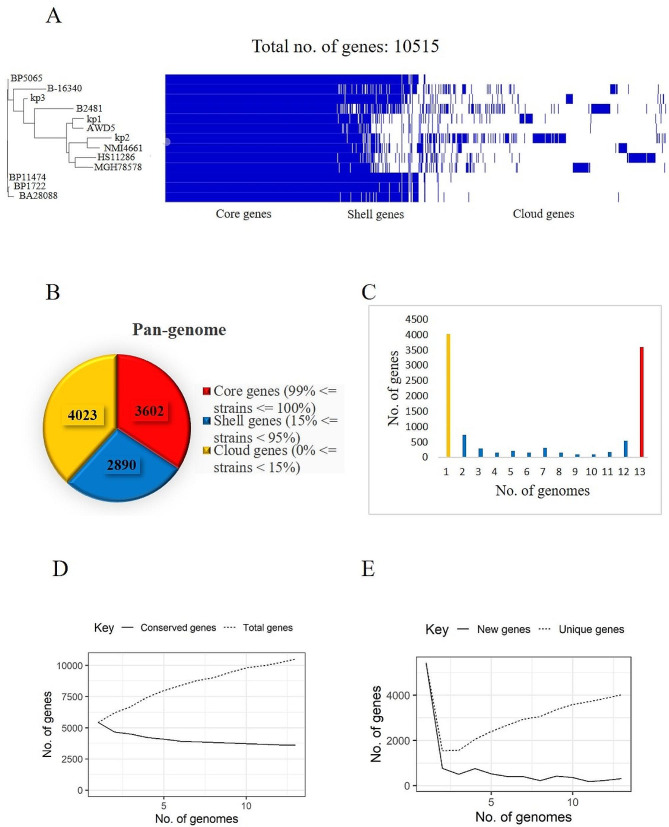
Pangenomic analysis of *K. pneumoniae*: (**A**) Heat Map representing core, shell and cloud genes among 3 study strains and 10 reference strains. (**B**) Pie chart representing the abundance of core, shell and cloud genes. (**C**) Histogram depicting the frequency of genes among *K. pneumoniae* strains. The red bar indicates core genes present in all the genomes (*n* = 13); the yellow bar represents unique/cloud genes. The blue bar represents the shell genes that are shared among the strains. (**D**) Heap’s law chart representation regarding conserved genes vs. total genes. The number of new (v) and conserved (iv) genes saturized with 13 genomes. The numbers of unique (v) and total (iv) genes were positively correlated with the number of genomes included in the analysis, as shown by a constant increase of identified genes when the number of genomes increased

**Fig. 3:**
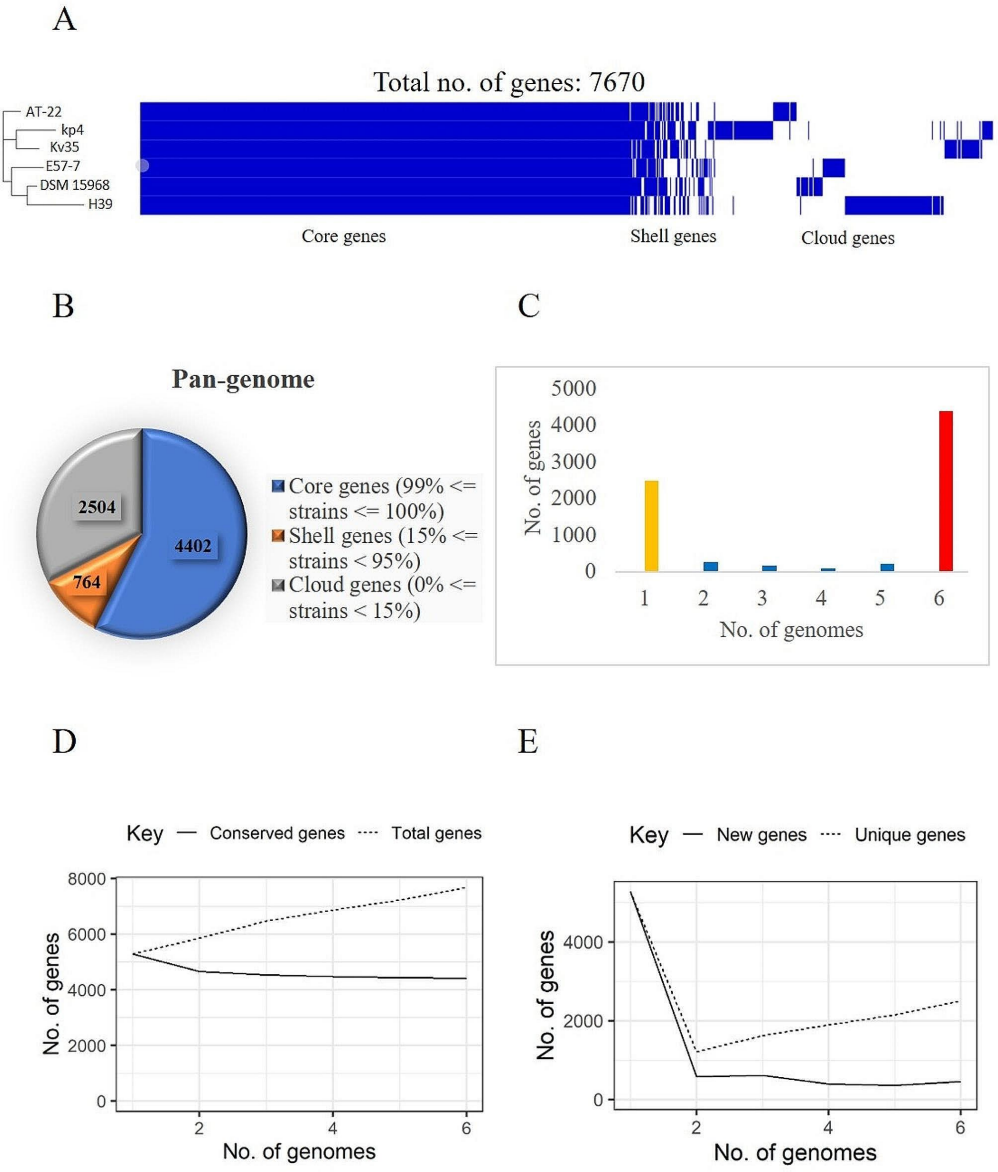
Pangenomic analysis of *K. variicola*: (**A**) Heat Map representing core, shell and cloud genes among 1 *K. variicola* study strains and 5 reference strains. (**B**) Pie chart representing the abundance of core, shell and cloud genes. (**C**) Histogram depicting the frequency of genes among *K. variicola* strains. The red bar indicates core genes present in all the genomes (*n* = 6); the yellow bar represents unique/cloud genes. The blue bar represents the shell genes that are shared among the strains. (**D**) Heap’s law chart representation regarding conserved genes vs. total genes. The number of new (v) and conserved (iv) genes saturized with 6 genomes. The numbers of unique (v) and total (iv) genes were positively correlated with the number of genomes included in the analysis, as shown by a constant increase of identified genes when the number of genomes increased

Pangenome analysis aids in understanding the genome architecture by providing information on core and accessory genes. The protein-coding genes in the 13 genomes which include 3 study strains and a few *K. pneumoniae* Indian isolates (*n* = 8) were around 10,515 genes. The core and accessory genome comprised 3602 and 6913 (about 66% in pan-genome) genes respectively including, shell genes (*n* = **2890**) and cloud genes (*n* = **4023**) in *K. pneumoniae* (Fig. 2). Similarly, the core, shell and cloud genes for *K. variicola* reference and study strains are 4402, 764 and 2504 respectively (Fig. 3). The number of accessory genes of kp1, kp2, kp3 and kp4 is 1338, 2489, 1770 and 1140 respectively. The approximate number of AMR genes carried in the accessory genome of kp1 (*n* = 49), kp2 (*n* = 53) and kp3 (*n* = 55). The acquired AMR genes include *bla*_SHV−1_, *bla*_OXA−10_, *bla*_TEM_, *bla*_CTX−M−1_, *bla*_SHV−2_ and Metallo-β-lactamase type-2. Similarly, virulence genes (*n* = 37) were present in the core genome whereas the acquired virulence genes in kp1, kp2 and kp3 are 14, 32 and 15 respectively. This includes secretion system genes, fimbrial genes and siderophore genes. kp4 has 56 virulence genes in the genome, 8 in the accessory genome and 53 AMR genes. Among the study strains, kp2 has more strain specific and common genes. Collectively, 66% and 43% of the complete genome was comprised of the accessory genome in *K. pneumoniae* and *K. variicola* respectively. The pangenome analysis indicates high genome diversity in *Klebsiella* spp. due to the high frequency of gene mutation rather than horizontal gene transfer [Supplementary Table S2].

### Comprehensive AMR profiling reveals highly resistant genes in clinical isolates

Using CARD, Resfinder and PATRIC, the complete genomes of the four isolates were screened for AMR elements. Around 38 antibiotic inactivation genes were predicted and observed as the major category of antimicrobial resistance mechanism, followed by antibiotic efflux (*n* = 16 genes) and antibiotic target alteration (*n* = 7 genes) among the study strains. Among these, the commonly shared AMR genes in the genomes are efflux pump (*n* = 12), antibiotic inactivation (*n* = 1) and target alteration (*n* = 6). The common putative drug efflux systems majorly fall into the Resistance Nodulation Cell Division (RNCD) and Major Facilitator Super Family (MFS). The four isolates were detected with *oqxA*, *crp*, *rsmA, adeF, baeR, hns*, *marA* and *AcrAB* in the RND gene family. Similarly, *kpnF, kpnH, kpnG*, *emrR* of MFS gene family.

kp3 isolate was found to be harboured with a high AMR gene across a wide range of antibiotic classes. Majorly with *qnrB17, gyrA, gyrB* (Quinolone), *bla*_SHV−11_, *bla*_NDM−5_, *bla*_OXA−181_ (β-lactamase) *aac(6’)-Ib-cr6, aph(6)-Id, aph(3’’)-Ib, aadA* (Aminoglycoside), *ereA2*, *mphA* (Macrolide), *arr-2* (Rifamycin), *sul2, sul1* (Sulphanamide), *cmlA5* (Chloramphenicol), *dfrA14* (Trimethoprim), which conveys kp3 has more AMR genes and broader antimicrobial spectrum of genes than the other isolates. In addition, the kp3 isolate was harboured with antiseptic resistant gene *qacE*Δ*1*. The environmental strain *K. variicola* kp4 has the least number of AMR genes. *mcr-1* gene conferring resistance to colistin is absent in the study strains. The comprehensive AMR genes involved in various resistance mechanisms are provided in Supplementary Table S3.

### **Correlation of **in vitro **susceptibility test with resistome**

When tested against 11 antibiotics of β-lactams, kp1, kp2 and kp3 were almost resistant to all, whereas kp4 was sensitive to ceftazidime, ertapenem and imipenem. kp4 displayed resistance to 8 antibiotics but was detected with *bla*_LEN−16_ alone which is inherent to *K. variicola*, suggesting *bla*_LEN−16_ is not significantly associated with a genotypic marker of β-lactam resistance. The exhibited phenotypic resistance could be through antibiotic efflux or antibiotic target alteration. In the case of quinolone, all strains exhibited resistance to ciprofloxacin, norfloxacin, ofloxacin, which correlates with gene prediction that includes multiple mutations of the quinolone resistance-determining regions (*gyrA*, *gyrB*) and plasmid mediated quinolone resistance (*qnrb1*, *qnrb17*, *qnr10*).

Though the most commonly reported macrolide resistance genes *mef*, *mreA* were not predicted, kp3 alone carried *ereA2* and *mphA*. The other strains have efflux pump (*kpnE, kpnF, kpnG, kpnH, hns, crp*) genes. In concordance with the genomic profile, erythromycin and azithromycin resistance was observed in all the strains. Similarly, gentamycin and amikacin resistance were recorded for all 4 strains which harbour aminoglycoside inactivation and antibiotic efflux genes. Phenotypic chloramphenicol resistance was observed for the kp3 strain alone which correlates with the presence of the *cmlA5* gene. Similarly, the absence of chloramphenicol resistance genes was correlated with phenotypic sensitivity. In all the studied strains 100% susceptibility to colistin was observed in correlation with the absence of colistin resistance genes (*mcr-1*). From the genotype and phenotypic determination of the resistance pattern, all the isolates were found to be Extensive drug resistance (XDR) (Magiorakos et al. 2012). A high MAR index indicated that the isolates are considered to be resistant (Table 1). In summary, the in vitro and genomic AMR profiling correlated well for all the tested antibiotics against the study strains (Table 2).

**Table 1:** MAR index for the study isolates

Strain Id	MAR index
Kp1	0.875
Kp2	0.8125
Kp3	0.9375
Kp4	0.6875
Average (aggregate score)	0.828125

**Table 2:** Resistome and phenotypic correlation of AMR

Antimicrobial agent	*Antibiotic susceptibility test				Presence of Genes (comprehensive analysis)			
	kp1	kp2	kp3	kp4	kp1	kp2	kp3	kp4
**Penicillin** Ampicillin Oxacillin Methicillin	R R R	R R R	R R R	R R R	*bla*_SHV−134_ *bla*_SHV−12_	*bla*_SHV−1_ *bla*_CTX−M−15_ *bla*_SHV−26_ *bla*_SHV−199_ *bla*_SHV−194_ *bla*_SHV−78_ *bla*_TEM−1 A_ *bla*_SHV−145_ *bla*_SHV−98_ *bla*_SHV−179_	*bla*_SHV−11_ *bla*_CTX−M−15_ *bla*_SHV−67_ *bla*_OXA−1_ *bla*_OXA−181_ *bla*_SHV−11_ *bla*_NDM−5_ *bla*_TEM−1B_	*bla*_LEN−19_ *bla*_LEN−16_
**First generation** Cefazolin Cefadroxil	R R	R R	R R	R R				
**Second generation** Cefuroxime	R	R	R	R				
**Third generation** Cefepime Cefpodoxime Ceftazidime	R R R	R R R	R R R	R R S (2 µg/mL)				
**Carbapenem** Ertapenem Imipenem	R R	S (0.125 µg/mL) R	R R	S (0.125 µg/mL) S (0.5 µg/mL)				
**Quinolone** Ciprofloxacin	R	R	R	R	*gyrA* *gyrB*	*qnrB1* *qnrB10* *gyrA* *gyrB*	*qnrB17* *qnrB10* *gyrA* *gyrB*	*gyrA* *gyrB*
**Macrolide** Erythromycin	R	R	R	R	*kpnE* *kpnF* *kpnG* *kpnH* *hns* *crp*	*kpnE* *kpnF* *kpnG* *kpnH* *hns* *crp*	*ereA2* *mphA* *kpnE* *kpnF* *kpnG* *kpnH* *hns* *crp*	*kpnE* *kpnF* *kpnG* *kpnH* *hns* *crp*
**Aminoglycoside** Gentamycin	R	R	R	R	*aac(3)-IIe* *kpnE* *kpnF* *kpnG* *kpnH* *baeR*	*kpnE* *kpnF* *kpnG* *kpnH* *baeR*	*aac(6’)-Ib-cr6* *aph(6)-Id* *aph(3’’)-Ib* *aadA* *kpnE* *kpnF* *kpnG* *kpnH* *baeR*	*aadA* *kpnE* *kpnF* *kpnG* *kpnH* *baeR*
Chloramphenicol	S (2 µg/mL)	S (2 µg/mL)	R	S (2 µg/mL)	Absent	Absent	*cmlA5*	Absent
**Polymyxin** Colistin	S (0.5 µg/mL)	S (0.5 µg/mL)	S (0.5 µg/mL)	S (0.5 µg/mL)	Absent	Absent	Absent	Absent
**Type of Resistance**	XDR	XDR	XDR	XDR	XDR	XDR	XDR	XDR

*Tested using ABST and MIC, R – Resistant, S – Sensitive

### Episome of kp3 harbours β-lactam, aminoglycoside and tetracycline resistant gene

Except for kp1, other strains were found to have at least three replicon types associated with their genome. The Inc group plasmid was dominantly present. The AMR analysis of episome revealed the presence of *bla*_NDM−5_, *bla*_KPC−3_, *bla*_CTX−M−15_, *bla*_NDM_, *qnrB1, bla*_OXA−1_, *bla*_TEM−1_, *bla*_KPC−2_ [Supplementary Table S4 (Sheet 1)]. When compared with other strains, kp3 was found to have plasmids encoding β-lactam, aminoglycoside and tetracycline resistant genes. The *K. variicola* (kp4) was also found to have pBK30683, pNDM-MAR, pK2044, pKPN3 harbouring β-lactamase and tetracycline resistant genes.

### Transposons

The strains were harboured with eleven insertion sequence elements such as IS*1*, IS*110*, IS*200*/IS*605*, IS*21*, IS*3*, IS*30*, IS*630*, ISL*3*, ISNCY, Tn*3* and IS*1182*. Among these, kp3 was found to have a greater number of IS elements (*n* = 9). In contrast, kp2 was found to have a single IS element [Supplementary Table S4 (Sheet 2)]. Briefly, these IS elements carry genes conferring resistance to tetracycline, chloramphenicol and aminoglycoside (Vapnek and Kirby Alton 1980; Gawryszewska et al. 2017). Collectively, the AMR elements found in the genome, episome and the number of IS elements were found to be higher in kp3 than the other strains and that was also evident in the antimicrobial susceptibility test.

### Absence of AMR genes in prophages

A total of 9 prophage elements were detected. The total size of the prophage genome ranges from 42.2 kb (kp1) to 106.4 kb (kp3). The average GC content of the prophages integrated among the 4 strains was 53.87%, suggesting the transduction of the prophage region. The prophage comprised 1-1.8% genome size of the study isolates. Among these, 3 prophages belong to *Myoviridae* and 6 prophages to *Siphoviridae*. kp3 strain has the highest number of prophages (*n* = 6) including intact and cryptic or defective phages. The clinical strains have one intact prophage each, whereas the environmental strain has 3 cryptic prophages, that are reported to offer several advantages to the host (Ramisetty and Sudhakari 2019). The annotation of these intact prophages demonstrates the detection of structural and regulatory genes. The genomic analysis of the prophage genome reveals the proteins involved in the transporter, replication, structural proteins, recombination, host cell lysis and heat shock proteins. The lysis genes involved in bacterial cell lysis such as endolysin, holing and spanin were also detected. Endolysin genes were detected in both intact and cryptic phages. One of the cryptic kp3 prophages was found to have IS*1* transposase and Tn*9*. None of the isolates have AMR genes in the phage genome [Supplementary Table S4 (Sheet 3)].

### **Association of biofilm virulome and **in vitro

The fimbriae, capsule synthesis, adherence, colibactin, iron uptake, magnesium uptake and biofilm formation genes were identified. All the isolates were found to have type I and III fimbriae that aid in adhesion to host cells and medical devices. Type IV pili have an important role in twitching motility and adherence was present only in kp1 and kp3 genomes. kp3 has major fimbrial determinants encoded by *ste, stf* and kp4 strain with *stc* and *sti* fimbrial operons of *Salmonella enterica* serotype Typhimurium. These operons are involved in adhesion, colonization and pathogenesis (Forest et al. 2007). This is the first report on *Klebsiella* spp. having fimbrial operons of *Salmonella* [Supplementary Table S4 (Sheet 4)].

**Fig. 4:**
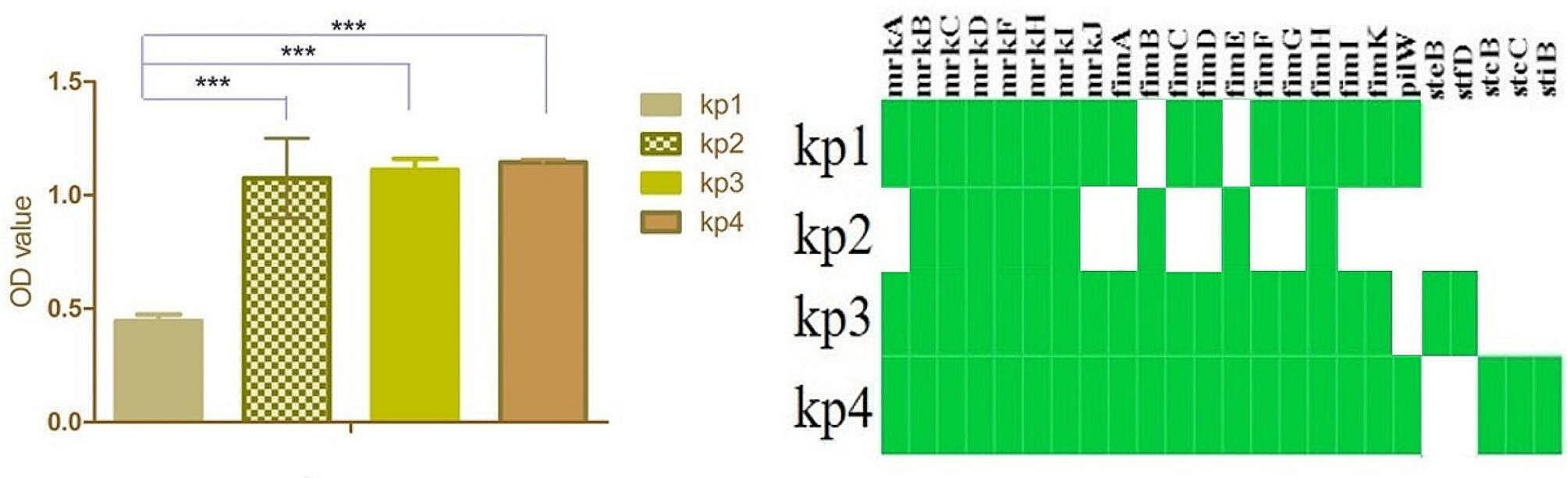
Comparison of biofilm formation: (**A**) Biofilm formation ability among the study strains. *** Indicates *p* < 0.0001; kp1 forms significantly less biofilm than the other strains; (**B**) Heatmap represents the presence/absence of biofilm related genes

The proficiency of biofilm formation of the strains was determined to correlate with biofilm related genes. The kp1 forms significantly less biofilm (OD: 0.08) than the other three strains (kp2: OD 0.9, kp3, 1.07, kp4: 1.1) on the abiotic surface (Fig. 4). Though all the relevant genes were found in all the strains, the reason behind the less biofilm development in kp1 is not known clearly.

To identify the hypermucoviscosity phenotypes, the regulatory genes *rmpA* and *magA* were analysed. All the study strains were completely absent of the hypermucoid phenotype gene (Supplementary Fig. S3). Based on the sedimentation test kp3 is significantly found to be more mucoviscous when compared to other strains (Supplementary Fig. S4). Besides, *RcsAB*, a two-component regulatory system that regulates capsular polysaccharide biosynthesis was found suggesting the presence of capsule in all the strains. The in vitro validation test was found to be negative and concordant with the absence of hypermucoviscosity genes (*rmpA* and *magA*). The presence of mucoid colonies and capsules were noted for the strains.

The prevalence of the four most important siderophore systems in Enterobacteriaceae are yersiniabactin (*ybtS*), aerobactin (*iutA*), enterobactin (*entB*) and salmochelin (*iroE*) were screened. The *entB, iutA*, *iroE* and *iroN* genes were detected in all the genomes, whereas *ybtS* gene was found in the kp2 and kp3 genomes alone. In addition, the allantoin utilization (*allS*) gene associated with hypervirulence was not detected in any of the strains (Shon et al. 2013). With this backdrop, the in vivo virulence characterization was performed.

### **Absence of correlation between virulome and **in vivo **pathogenicity**

**Fig. 5:**
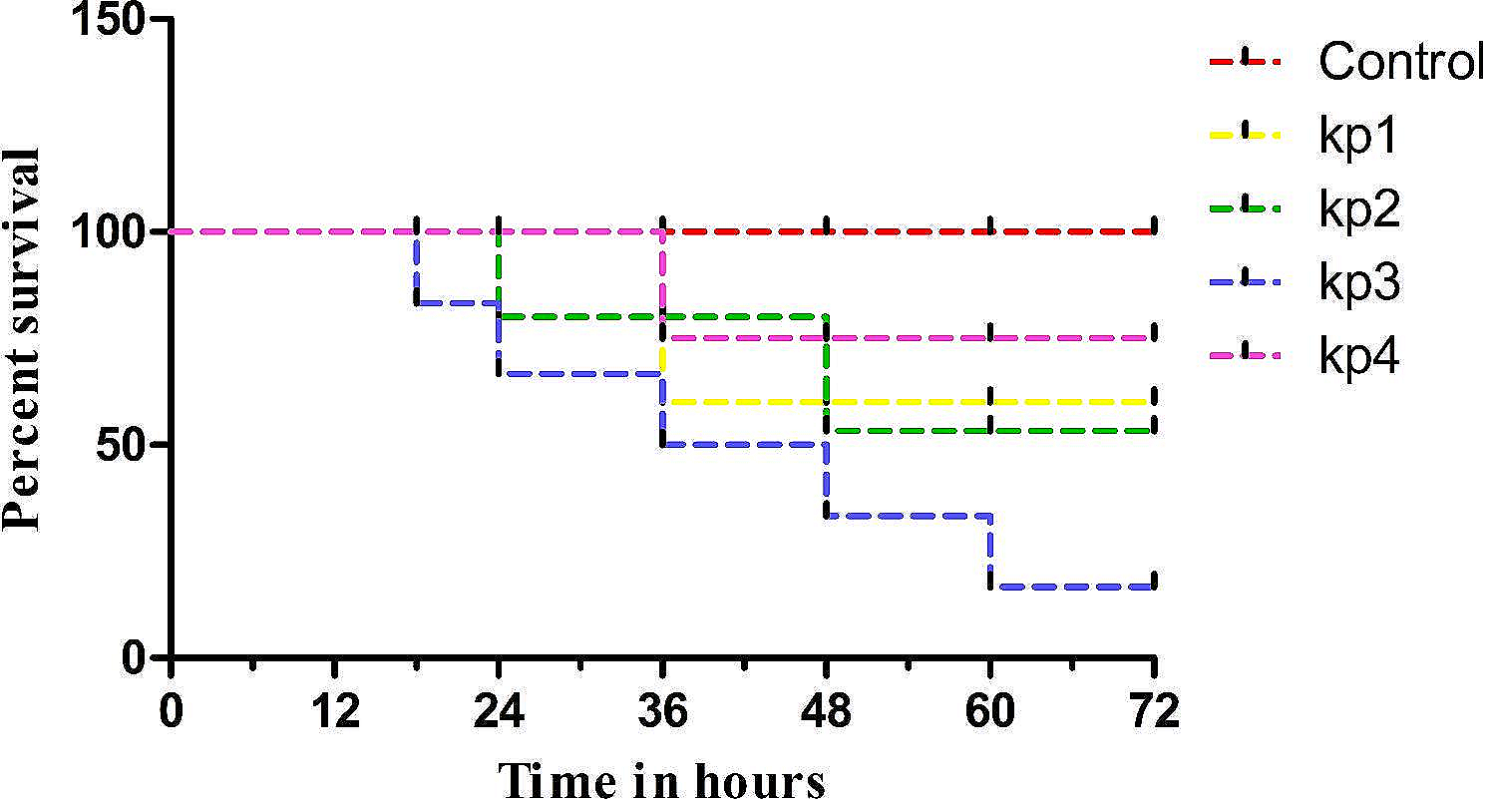
In vivo virulence characterization in Zebrafish infection model: Survival curve of four infected groups. The blue line represents the statistically significant decreased survival of Zebrafish infected with kp3. The experiment were repeated twice on two independent days (*n* = 7). *P* < 0.0001 signifies that the kp3 infected group exhibits more mortality

**Fig. 6:**
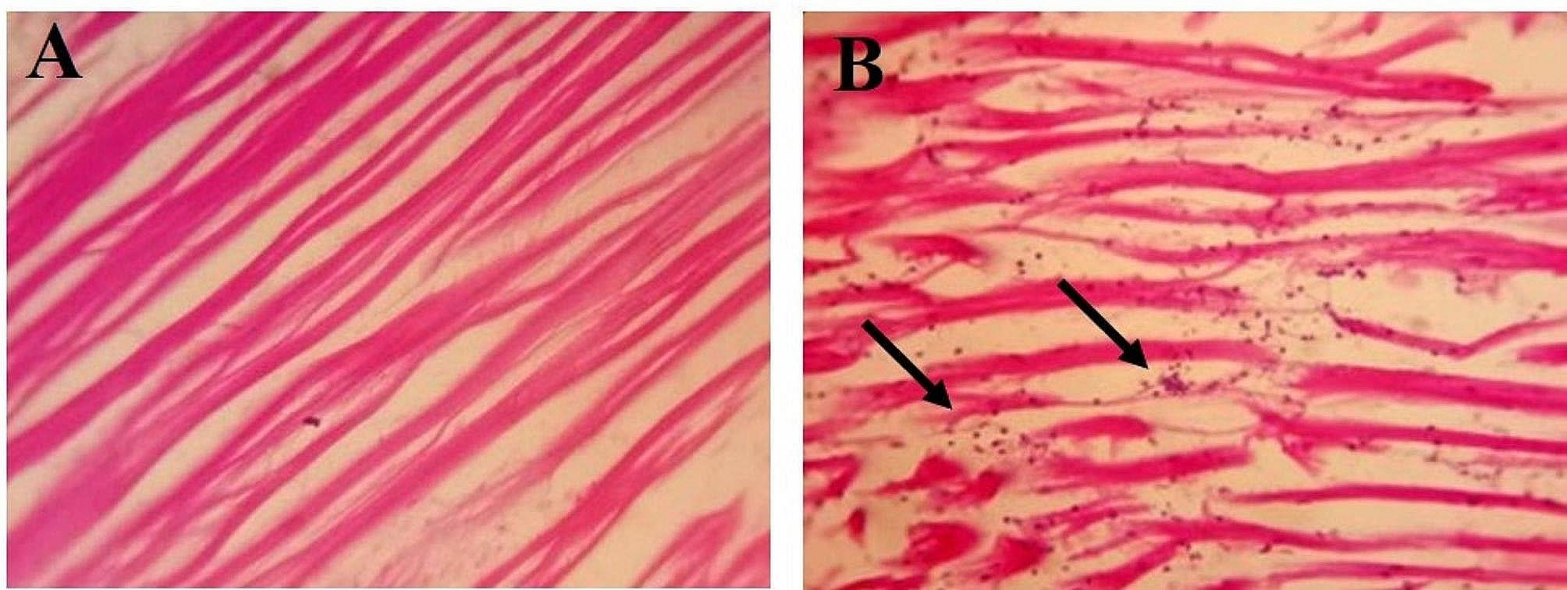
Histopathology of infected fish muscle: (**A**) Control, (**B**) Infected with kp3 isolate; arrow indicates the cell infiltration due to invasiveness of infection

Likewise, with AMR genomic profiling and in vitro assay, the virulome and virulence were studied using Zebrafish as an in vivo model. When the adult immune-competent fish were challenged with 10^8^ CFU/ml no clinical symptoms were observed in any of the strains. Whereas, a clinical dose of 10^12^ CFU/ml leads to infectious symptoms (reduced motility, feed intake) and 71% of mortality was observed for kp3 within 72 hpi. Other isolates were found to have less than 30% of mortality (Table 3), (Fig. 5). We then analysed the histopathology of kp3 infected fish muscle, when compared with control (uninfected), kp3 infected fish was found to have cell infiltration suggests the invasiveness of kp3 (Fig. 6). While investigating the virulence genes, especially fimbrial adherence genes, kp3 and kp4 harbours all the listed genes (n-18), whereas kp1 and kp2 have 16 and 9 genes respectively. Similarly, Fimbrial adherence determinants of *Salmonella* species are found in kp3 (*ste, stf*) and kp4 (*stcB, stcC, stiB*) alone. *rmpA* and *magA* genes contributing to the hypermucoid phenotype is absent in all the study strains. Though the virulence genes harboured by kp3 and kp4 are similar, the mortality percentage (71% and 28% respectively) of these two strains varied. Suggesting the discordance of virulome and virulence in *Klebsiella* spp.

**Table 3:** Estimation of *Klebsiella* burden in deceased Zebrafish

Group	No. of fish	Klebsiella burden in deceased fish muscle	Mortality percentage
kp1	7	1.06 × 10^9^	28.6%
kp2	7	4.40 × 10^8^	28.6%
kp3	7	8.12 × 10^8^	71.43%
kp4	7	1.04 × 10^8^	28.6%
Control	7	0	-

## Discussion

Though our first report on the comparative genomic study of Indian isolates of *K. pneumoniae* collected during 2010–2020, characterized the convergence of resistance and virulence in *K. pneumoniae* as a major concern, the correlation of phenotype and genotype remains scarce for *K. pneumoniae*. High virulence and PDR in *K. pneumoniae* lead to treatment failure, recurrent infections and morbidity. Hence, it’s a critical need to understand the genotypic and phenotypic correlation of *Klebsiella* spp. to improve the diagnosis of the pathotypes and treatment.

**Fig. 7:**
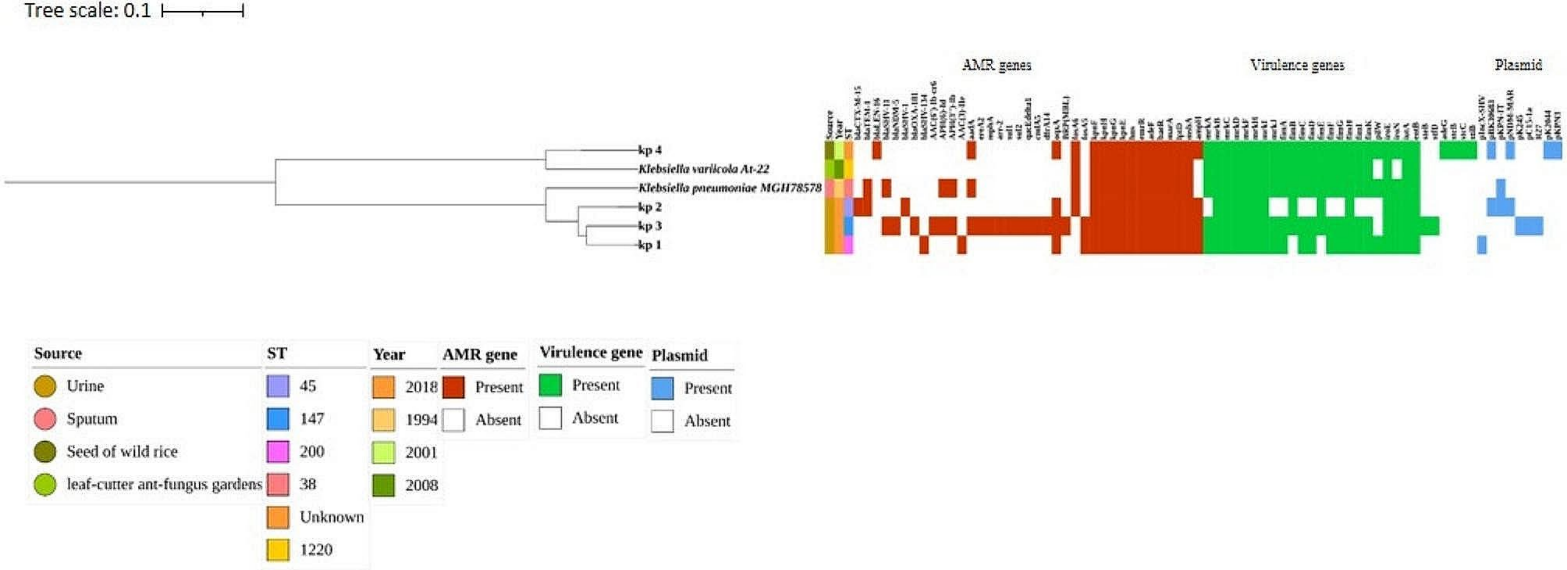
A complete picture of SNP-based phylogeny of the study strains: The isolation year, source and serotype are indicated by different colours. The presence of AMR genes, virulence genes and replicon types are indicated with red, green and blue respectively

The hybrid sequencing of the 4 strains using Illumina and Nanopore reads together provided complete genome data that overcame the limitations in terms of contig length. (Supplementary Table S1). As WGS is growing worldwide for the prediction of species, AMR elements and resistance mechanisms, a correlation analysis of genetic and phenotypic is required to identify and track high-risk pathogens. We examined the STs, serotypes, pangenome, antimicrobial resistance, transposons, virulence, replicon types and integrated phage genome analysis of the study strains to conduct phenotype/genotype correlation studies (Fig. 7).

*Klebsiella* spp. harbours almost all the resistance genes that are resistant to most of the antibiotics. They use various strategies to confer resistance to the existing antibiotic drugs. The predominant resistance mechanism is the enzymatic degradation of a particular class of antibiotics (β-lactam) by producing β-lactamase. Other mechanisms are antibiotic target alteration, changes in membrane permeability and efflux pumps. The resistance conferring enzymes are acquired by mobile genetic elements like plasmids and transposons (Moya and Maicas 2020; Huy 2024; Li et al. 2024). In the present study, *bla*_SHV_ was commonly found in all the uropathogenic *K. pneumoniae* and is resistant to clavulanic acid. kp3 strain alone possesses Amber class A, B, and D β-lactamases, indicating the broad spectrum of activity against penicillins, cephalosporins and carbapenem, whereas the other strains have Class A enzyme alone. The *K. variicola* (kp4) was found to be susceptible to the tested carbapenems. The clinical kp2 was noted to have discordance in susceptibilities between ertapenem (sensitive) and imipenem (resistant), this is in line with the previous report (Andrew Chou, Haley Pritchard, Richard Sucgang 2017). While comparing the comprehensive resistome with susceptibility tests, we observed 100% concordance for β-lactam, quinolone, aminoglycosides, macrolide, Chloramphenicol and Colistin. Some of the previous reports showed concordance (Devanga Ragupathi et al. 2020; Lam et al. 2021) and some stated the discrepancy between genotype and phenotype AMR correlation (Lomonaco et al. 2018; Urbaniak et al. 2018). In the present study, we observed no disagreement for the tested broad-spectrum antibiotics. Hence the present study suggests that WGS is a suitable tool for rapid detection of AMR patterns. However, a comprehensive AMR genomic analysis is required for accurate assessment by characterizing the presence/absence of key genes involved in resistance mechanisms of all antibiotic classes that would further facilitate the use of WGS in clinical trials and improve patient care.

The capsular polysaccharide is a prominent virulence factor in *K. pneumoniae* responsible for the evasion of the host immune system. K1/K2 capsular serotypes are common to both hvKP, cKP (Shon et al. 2013). Some of the studies reported that serotypes and virulence are not directly correlated. For instance, in our previous report on comparative genome analysis, the hypervirulent associated *rmpA* and *magA* genes were present in non-K1/K2 strains as well (Sundaresan et al. 2022). In the present study, we identified 3 novel K-loci serotypes and an O serotype among the studied strains. The K-loci of 3 strains include a common set of genes (*galF*, *wzi*, *wza*, *wzb*, *cpsACP*, *gnd* and *ugd*) required for capsule biosynthesis and *K. variicola* (kp4) was found to lack *galF*, *gnd, ugd* and *cpsACP*, suggesting the variation of core genes of capsule biosynthesis in *K. pneumoniae* and *K. variicola.*

Prophage region was identified in all the genomes, an important element for genome plasticity and evolution (Ramisetty and Sudhakari 2019). Intact phage was found in all *K. pneumoniae* strains whereas *K. variicola* has cryptic phage. The presence of intact phage is evident for recent integration. As in line with previous reports, *Klebsiella* phages belong to *Mycoviridae* and *Siphoviridae* (Marques et al. 2021). In both cryptic and intact prophage, the AMR genes and virulence genes were not found, suggesting the utility of phage for phage therapy. We also noticed a high number of hypothetical proteins in the genome conveying the scarcity in understanding phage protein functions before considering its utility for phage therapy.

Phenotypic virulence traits of the study strains were observed by in vitro pathogenicity tests. The negative string test for all the strains is correlated with the absence of *rmpA* and *magA* genes.

Biofilm formation by the pathogen is associated with chronic infections and acts as a physical barrier in protecting the pathogen from the host immune system. For the formation of biofilm, initially, the genes encoding the components of type I fimbriae (*fimA, fimB, fimC, fimD, fimE, fimF, fimG, fimH, fimI, fimK*) are involved in establishing the attachment of bacteria to the surface and type III fimbriae (*mrkA, mrkB, mrkC, mrkD, mrkF, mrkH, mrkI, mrkJ*) are involved in forming biofilm on the abiotic surface such as indwelling devices. Expression of *rmpA* a regulator of mucoid phenotype enhances the biofilm formation. During the biofilm formation, the aggregate of microorganisms produces an Exo-polymeric matrix which ensures that the bacteria is less susceptible to antibiotics (Zheng et al. 2018; Nirwati et al. 2019). In our phenotypic study, the biofilm formation was also converging with the representation of biofilm related genes. In the Zebrafish virulence study, the kp3 strain alone caused 71% of Zebrafish death when infected with 10^12^ CFU/ml. Infection assay with other strains (kp1, kp2, kp4) resulted in very low mortality (28.6%, 28.6%, 28.6%). The virulome analysis depicted the absence of *rmpA*, *magA* and *allS* which are important virulence determinants in hvKP.

The hypervirulent traits converge with in vitro and in vivo methods. This is in line with a previous report where low *rmpA* expression levels contributed to the absence of hypervirulent phenotype of *K. pneumoniae* in the mouse infection model (Lin et al. 2020). Similarly, the *magA* mutants that lost hypermucoviscosity exhibited avirulence in the mice infection model (Lin et al. 2011). There is limited virulence characterisation of *K. variicola* in the mice infection model.

Though the important hypervirulence determinants are absent in all the studied strains, the high mortality caused by kp3 might be due to the presence of *ste, stf* fimbrial operons, that mediate diverse functions like adhesion, colonization, biofilm formation and pathogenesis. The kp4 predicted with *stc* and *sti* fimbrial operons was observed with 28% mortality in Zebrafish. Hence, we suggest the presence of *ste, stf* fimbrial operons in kp3 might contributed to high virulence in the Zebrafish infection model.

The summary of genomic analysis among the tested strains depicted that kp3 has a broad spectrum of antimicrobial resistance having a high number of episome, IS elements and intact phages that could have also contributed to its multidrug resistance. kp3 displayed mortality in the Zebrafish infection model. These results collectively conclude that kp3 of ST147 is a multidrug resistant and virulent uropathogen. Though kp3 and kp4 share a common virulome, it is highly distinct in the Zebrafish infection model. Overall, the study highlights the concordance of genomic and phenotypic AMR profiling. CGS enhances the better understanding of mechanisms involved in drug resistance. However, a comprehensive AMR analysis is required for its usage in clinical practice. Similarly, the genomic and phenotypic marker of virulence is critically needed to diagnose and track the hypervirulent clones of *K. pneumoniae* among the complex *Klebsiella* spp.

### Electronic supplementary material

Below is the link to the electronic supplementary material.

Supplementary Material 1

Supplementary Material 2

Supplementary Material 3

Supplementary Material 4

Supplementary Material 5

Supplementary Material 6

Supplementary Material 7

Supplementary Material 8

Supplementary Material 9

Supplementary Material 10

## Data Availability

The datasets generated during and/or analysed during the current study are available in the NCBI repository under BioProject PRJNA650119. *K. variicola* MTCC 4030 was purchased from Microbial Type Culture Collection, India. kp1, kp2 and kp3 of *K. pneumoniae* strains were deposited at National Centre for Microbial Resource under the accession number MCC5428, MCC5427 and MCC5426 respectively.
