# Supplementary figures and images for: Complete genome sequence, phenotypic correlation and pangenome analysis of uropathogenic Klebsiella spp
Source: AMB Express. 2024 Jul 4;14:78. doi: 10.1186/s13568-024-01737-w (PMC11224175; doi:10.1186/s13568-024-01737-w)

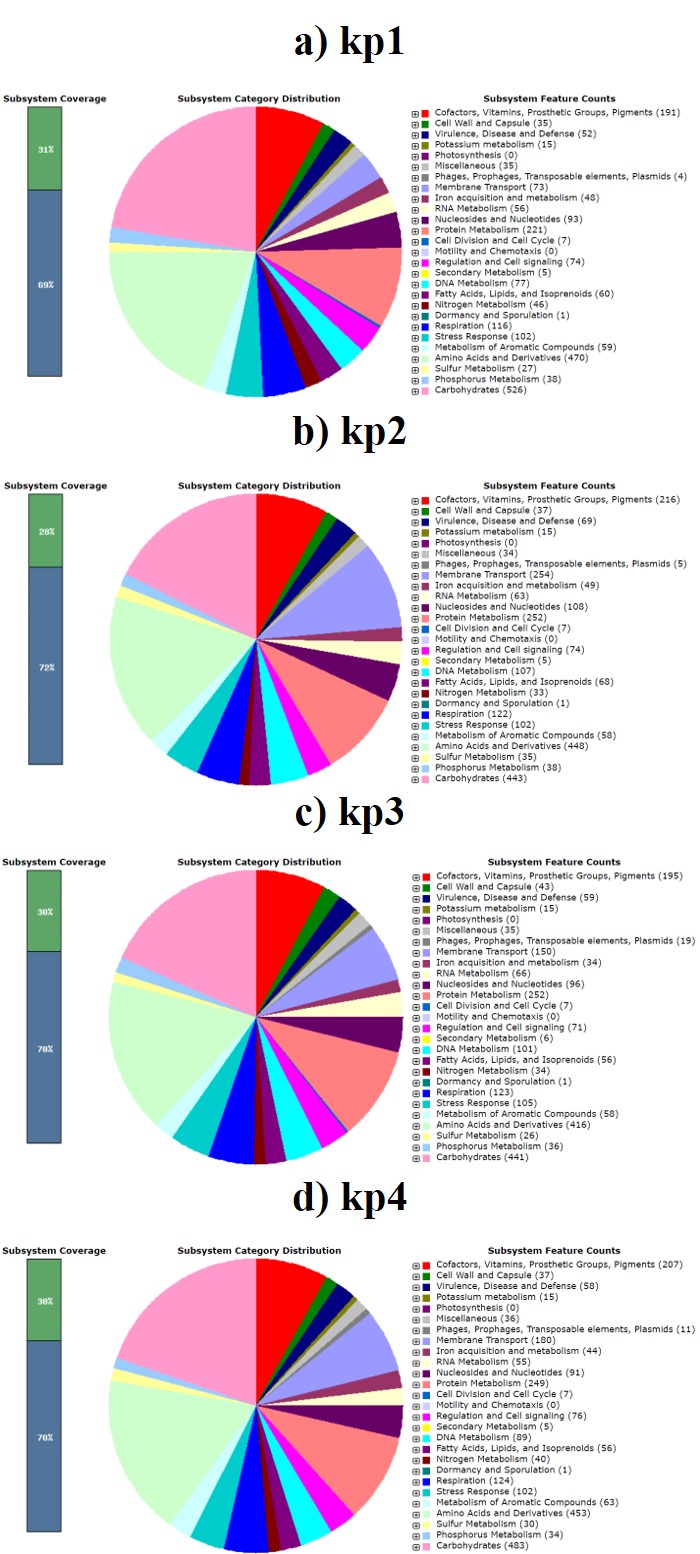

Supplement: Supplementary file 1 — Supplementary Material 1 [file 13568_2024_1737_MOESM1_ESM.jpg]

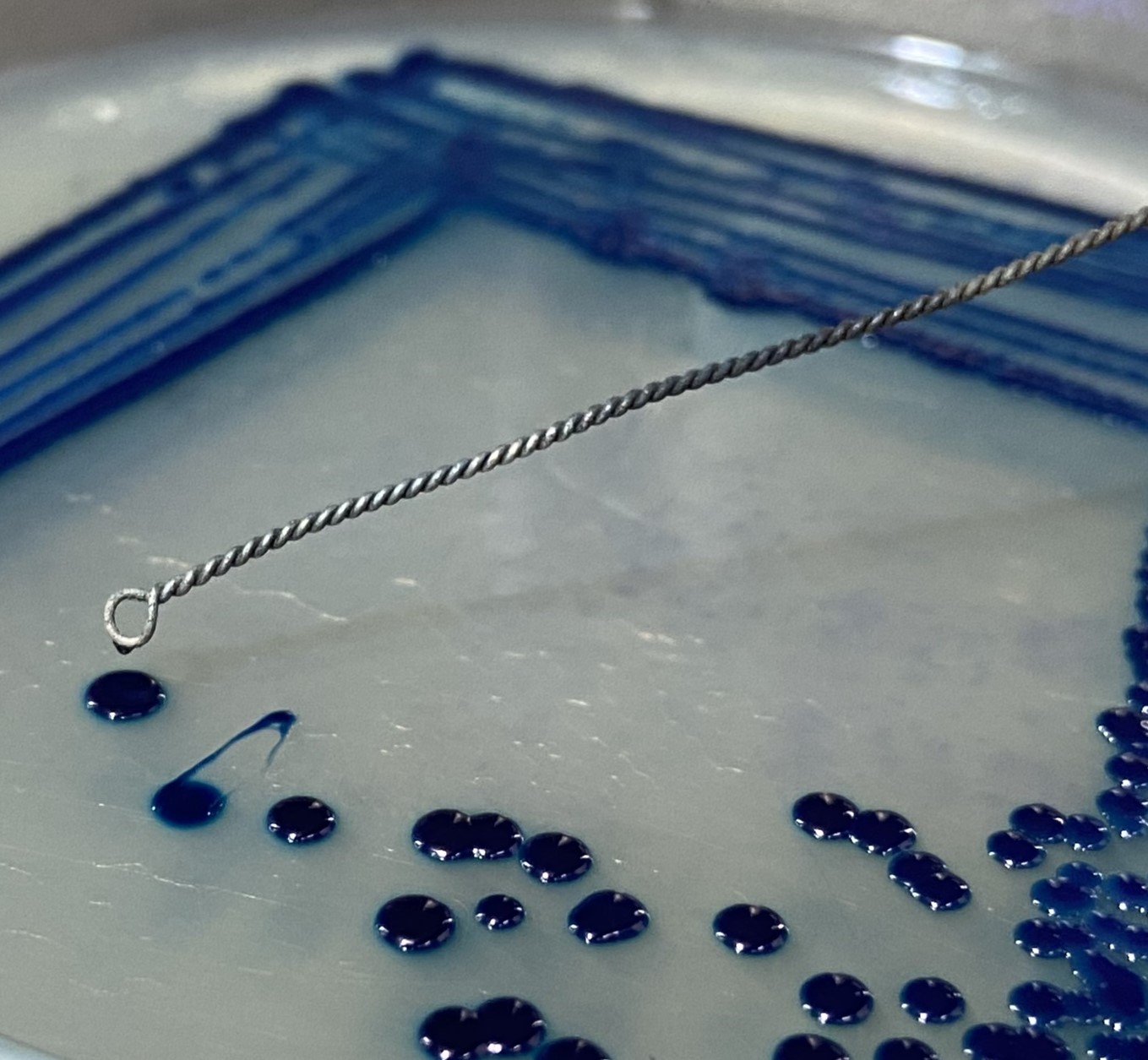

Supplement: Supplementary file 3 — Supplementary Material 3 [file 13568_2024_1737_MOESM3_ESM.jpg]

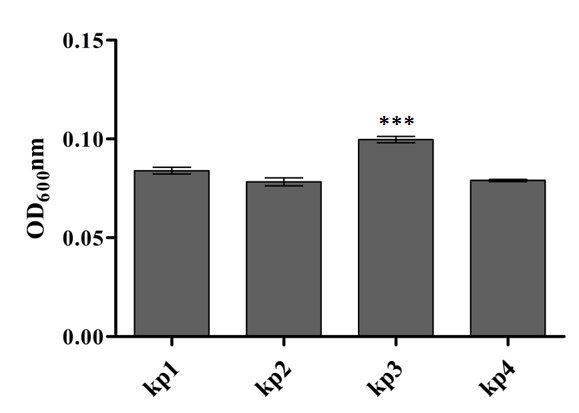

Supplement: Supplementary file 4 — Supplementary Material 4 [file 13568_2024_1737_MOESM4_ESM.jpg]
