# Supplementary material for: Complete genome sequence, phenotypic correlation and pangenome analysis of uropathogenic Klebsiella spp
Source: AMB Express. 2024 Jul 4;14:78. doi: 10.1186/s13568-024-01737-w (PMC11224175; doi:10.1186/s13568-024-01737-w)
Supplement: Supplementary file 5 — Supplementary Material 5 [file 13568_2024_1737_MOESM5_ESM.docx]

**Supplementary Methods**

**Procedure for library preparation and sequencing**

**Illumina library preparation and sequencing**

50 ng of Qubit quantified DNA was enzymatically fragmented, end-repaired and A-tailed in a one-tube reaction using the FX Enzyme Mix provided in the QIASeq FX DNA kit. The end-repaired and adenylated fragments were subjected to adapter ligation, whereby index-incorporated Illumina adapter was ligated, to generate sequencing libraries. These libraries were subjected to 10 cycles of Indexing-PCR (Initial Denaturation at 98 ̊C for 20 sec, cycling (98 ̊C for 20sec, 60 ̊C for 30sec, 72 ̊C for 30sec) and final extension at 72 ̊C for 1min) to enrich the adapter-tagged fragments. The amplified libraries were purified using JetSeq Beads, quantified on Qubit fluorometer and the fragment size distribution analyzed on Agilent TapeStation 2200. The qualified libraries were equimolar normalized and pooled for multiplexed high throughput sequencing. The library was paired-end sequenced on HiSeqXTen applying a read-length chemistry of 150 cycles. The sample was demultiplexed for the specific barcode used during library preparation, using bcl2fastq v2.0 tool.

**Nanopore library preparation and sequencing**

600ng of Qubit quantified DNA was end-repaired (NEBnext ultra II end repair kit, New England Biolabs, MA, USA), cleaned up with 1x AmPure beads (Beckmann Coulter, USA). Native barcode ligation was performed with NEB blunt/ TA ligase (New England Biolabs, MA, USA) using NBD104 and NBD114 (ONT) and cleaned with 1x AmPure beads. Qubit quantified barcode ligated DNA samples were pooled at equimolar concentration and Adapter ligation (BAM) was performed for 15 minutes using NEBnext Quick Ligation Module (New England Biolabs, MA, USA). Library mix was cleaned up using 0.6X AmPure beads (Beckmann Coulter, USA) and finally sequencing library was eluted in 15 μl of elution buffer and used for sequencing

Sequencing was performed on GridION X5 (Oxford Nanopore Technologies, Oxford, UK) using SpotON flow cell R9.4 (FLO-MIN106) in a 48 hrs sequencing protocol on MinKNOW 2.1 v18.08.3. Nanopore raw reads (‘fast5’ format) were basecalled (‘fastq’ format) and demultiplexed using Guppy basecaller v2.3.5.

**Biofilm Formation**

*K. pneumoniae* strains were separately grown overnight in LB broth at 37°C. The cells were then collected by centrifugation at 5,000 × g for 10 min, washed with PBS and resuspended in 10 ml of LB broth. The 96 well plate is inoculated with 500 μl (0.08 OD) of bacterial suspension, and incubated at 37°C for 72 h. The non-adherent cells were removed and biofilm were quantified by Crystal Violet assay (CV). All experiments were performed in duplicates or triplicates on two independent days.
