# Supplementary material for: Complete genome sequence, phenotypic correlation and pangenome analysis of uropathogenic Klebsiella spp
Source: AMB Express. 2024 Jul 4;14:78. doi: 10.1186/s13568-024-01737-w (PMC11224175; doi:10.1186/s13568-024-01737-w)
Supplement: Supplementary file 10 — Supplementary Material 10 [file 13568_2024_1737_MOESM10_ESM.docx]

**Supplementary Files**

**Supplementary Fig. S1: Subsystem categories**: Distribution of genes (cell wall and capsule, virulence, transposable elements, phages, cell division and cell cycle, metabolism) for four strains by RAST server.

**Supplementary Fig. S2: Phylogenetic tree of Indian isolates**: Phylogenetic tree for the study genomes were constructed with the previously studied Indian genomes of *Klebsiella pneumoniae* and reference *Klebsiella variicola* genome.

**Supplementary Fig. S3: String test**: Absence of hypermucoviscosity.

**Supplementary Fig. S4: Sedimentation method:** The hypermucoviscous cells in the supernatant was measured at OD_600_ after low-speed centrifugation of 1 OD_600_ unit of cells suspended in 1 ml of PBS. All the error bars denote standard deviation. *** indicates p < 0.0001. The experiment was carried out in triplicates.

**Supplementary Table S1 - Sequencing details** **of Isolates**- Metadata of the study strains, Nanopore read statistics, Comparison of Short reads, Long read and Hybrid assembly.

**Supplementary Table S2 - Pangenome data** - Details of Core, shell and cloud genes of *K. pneumoniae* and *K. variicola*

**Supplementary Table S3 - AMR profiling –** Results of AMR gene detection using CARD, ResFinder and PATRIC

**Supplementary Table S4 – Virulence and accessory gene prediction –** Virulence genes, episome, Insertion sequence and phages details were presented in the table
